# Characterisation and Risk Assessment of Metal Contaminants in the Dust Fall in the Vicinity of a Construction Waste Dump in Beijing

**DOI:** 10.3390/ijerph192013019

**Published:** 2022-10-11

**Authors:** Lili Wang, Gaofeng Wu, Tianyue Zhang, Wenkai Lei, Xinyu Wang, Mi Wang, Dongyang Zheng, Wenji Zhao

**Affiliations:** College of Resource Environment and Tourism, Capital Normal University, Beijing 100048, China

**Keywords:** construction waste, dust fall, heavy metals, geo-accumulation index, potential ecological risk index

## Abstract

In this study, a large construction waste dump in Beijing, China, was used as the study area. Nineteen effective atmospheric dust samples were collected. The mass fractions of 14 metal elements (Ca, Fe, Al, Mg, Mn, Zn, Cr, Cu, V, Pb, Ni, As, Co, and Cd) were determined for the samples using ICP-MS. The pollutants and the potential ecological risk levels of 10 different heavy metals were evaluated using the enrichment factor, geo-accumulation index, and a potential ecological risk assessment method. The results showed that the Ca, Fe, Al, and Mg contents in the dust fall were considerably high and accounted for 98.81% of the total mass of the analysed metals. Cd and Zn were the main metal contaminants in the dust fall in the vicinity of the construction waste dump, followed by Cu and Mn. The Cd, Zn, Cu, and Mn contents in the construction waste had a significant impact on atmospheric pollution within 250 m of the dump. Moreover, Cd had the largest contribution to the comprehensive ecological risk posed by the heavy metals in the dust fall and was determined to be the primary ecological risk factor in the atmospheric environment in the vicinity of the construction waste dump.

## 1. Introduction

The construction activities associated with economic development and rapid urbanization in China have led to an increase in the country’s construction waste. The annual construction waste produced by China is 1.55–2.4 billion tons [1], which accounts for approximately 40% of its urban waste [2]. China utilises less than 5% of its construction waste. In contrast, Europe and the United States utilise 70%, and Japan and South Korea utilise 90% [2,3,4,5]. The construction waste in most countries is transported to the suburbs for open stacking or traditional landfilling without any treatment [6]. Thus, waste not only occupies extensive areas of land, but also affects soil and groundwater environments in the vicinity of the construction waste dump [7,8,9]. Moreover, during the long-term stacking of construction waste, a large amount of dust is released [10]. The toxic and harmful heavy metals in construction waste are easily dispersed in the air, resulting in harm to the atmospheric environment and human health. Cd can cause damage to human kidneys, bones, and can cause lung injuries [11]. As can cause diabetes and heart disease [12] and Zn is related to lung inflammation [13]. Therefore, the management of construction waste is considered to be a serious social problem. However, since construction waste is not considered hazardous, the air pollution linked to its various processes has remained largely ignored.

In recent years, the environmental hazards posed by construction waste have attracted considerable attention regionally and globally. However, researchers tend to only focus on the impact of construction waste dumps on groundwater and soil. There is also no quantitative analysis and research on the impact of construction waste on the atmospheric environment in the stacking process. For example, López and Lobo [14] performed a leachate monitoring experiment on construction waste landfill in Europe. The results showed that the leachate contained typical construction waste pollutants, such as different inorganic ions and metals. Weber et al. [15] conducted characterization experiments on leachate from residential construction waste; their results showed that the leachate mainly consisted of Ca and sulfate, and that the concentrations of Al, As, Cu, Mn, Fe, and sulfate exceeded the water quality standards. Roussat et al. [16] research showed that demolition waste can contain arsenic, fluoride, chromium, cadmium, sulfate, dissolved organic carbon (DOC), and polycyclic aromatic hydrocarbons. Somasundaram et al. [8] detected heavy metals, such as Cr, Hg, and Zn, in concrete and cement waste. They emphasized that the presence of heavy metals in construction waste is largely determined by the source of construction waste. The above studies show that some chemical substances in construction waste cause harm to the ecological environment and that environmental pollution caused by construction waste cannot be ignored. However, quantitative analyses of the impact of construction waste on the atmosphere in the process of stacking are still scarce. Therefore, it is necessary to study metal pollution caused by atmospheric dust fall from construction waste and to perform the necessary risk assessments.

In 2018, the State Council of China promulgated a pilot work plan for the construction of a ‘waste-free city’, which proposed the treatment and improved management of construction waste [17]. Beijing is the capital of China, and its air pollution has always been the focus of attention. As construction waste is a significant source of atmospheric dust fall in Beijing [18], its impact on the atmospheric environment requires urgent investigation. In this study, we provided the concentration data of 14 metal elements and the pollution characteristics data of 10 heavy metals in the atmospheric dust fall of a large construction waste dump in Beijing, China. Specifically, the purpose of this study was to: (1) Explore the composition of metal elements in construction waste dust fall; (2) study the pollution characteristics of 10 kinds of toxic and harmful heavy metals in construction waste dust fall; and (3) analyse the potential risks of toxic and harmful heavy metals to the atmospheric environment around the construction waste dump. This study could provide a scientific and effective basis for the precise control of construction waste and the control of atmospheric dust, present a data foundation for subsequent research on the chemical composition of atmospheric dust fall of construction waste, and have a positive impact on the construction of a “waste free city.”

## 2. Material and Methods

### 2.1. Overview of the Study Site

In recent years, Beijing, China, has implemented several projects, such as the reconstruction of shanty towns, which have resulted in considerable construction waste that varies in type, composition, and complexity. A construction waste dump located in Beijing was selected for this case study. The area of the dump is approximately 59,000 m^2^, with several piles of construction waste distributed across the dump. The largest pile of construction waste is located in the southwest region of the dump and is approximately 20 m high. The dump is approximately 800 m south of Village A, 700 m northwest of Village B, and 1 km east of River C (Figure S1). According to a field survey, the construction waste in the dump is primarily solid waste produced by the demolition of nearby residential buildings and is mainly comprised of broken bricks and stones, cement fragments, ceramic tiles, gypsum boards, rusted metals, muck, wood, and some synthetic decorative building materials. The surfaces of the piles are covered with two dust nets; however, some surfaces remain exposed.

Spring and autumn in the study area are short, windy and dry. Additionally, summers are hot and rainy, whereas winters are cold and dry [19]. Hourly data from China’s surface meteorological station (station ID: 54596) nearest to the study area (data source: https://data.cma.cn/, accessed on 30 September 2022) show that, during the sampling period (60 ± 2 days from 3 September to 3 November 2020), the average wind speed, average precipitation, average relative humidity, and average temperature corresponded to 1.82 m/s, 1.36 mm, 62.53%, and 15.5 °C, respectively.

### 2.2. Sample Collection and Analysis

#### 2.2.1. Sample Collection and Processing

According to the aerodynamic theory, fine atmospheric particles are prone to migrate and diffuse across great distances under the action of air flow, while dust tends to settle. The concentration of heavy metals in atmospheric fine particles is influenced by fuel combustion, regional transport of industrial emissions, and meteorological factors [20,21,22]. However, the dust fall settles easily and has relatively weak diffusion capacity. Moreover, the pollution remains close to the source, making it suitable for atmospheric monitoring of the regional environment. Atmospheric dust derived from construction waste is mainly coarse particulate matter, most of which settles near the pollution source. The concentration of falling dust in the process of spatial migration decreases with an increasing distance from the dust source and is not easily disturbed by other particulate pollution sources. In this study, the sampling method of dust fall was continuous to assess long-term regional dust raising and the sedimentation of air particulate matter. This sampling method limits the sampling error associated with measurement uncertainty in terms of time and space [23,24]. Thus, considering that heating in Beijing started on 15 November, which may affect the composition of the dust samples in the study area, the sampling time was in autumn (60 ± 2 days from 3 September to 3 November 2020).

The sampling of atmospheric dust in the vicinity of the construction waste dump was performed according to a standard gravimetric method for the determination of ambient air dust (GB/T 15265-94) [25]. A glass dust-removal cylinder, with a height of 30 ± 0.2 cm, an inner diameter of 14 ± 0.17 cm, and a flat and smooth bottom, was used to collect atmospheric dust. Dust collection tanks must be placed away from the road and industrial pollution sources and must avoid the shelter of tall buildings. No tall buildings or industrial pollution sources are present near the study site, and other pollution sources are relatively concentrated. Therefore, considering the distance from the construction waste dump, samples were collected according to the radial distribution method, within a 1 km radius (Figure S1). A total of 25 dust monitoring points were established, and one control point was established in a park to the west (upwind) of the dump (as roads and residential neighbourhoods surround the site); the park is lushly vegetated and free of motor vehicles. Dust fall samples were recovered from 19 monitoring points (including the control point). However, dust fall samples could not be recovered from monitoring points 19, 20, 21, 22, 23, 24, 25, and 26 due to serious damage to the dust removal cylinders.

Before sampling, 60 mL ethylene glycol was placed in each dust tank to inhibit the growth of algae and microorganisms, and 200 mL distilled water was added to keep the bottom of the tank moist and to reduce the impact of secondary dust. During transportation, the mouths of the cylinders were sealed with plastic film to prevent the dust generated during transportation from affecting the test results. The dust removal cylinders were checked every two weeks for damage and loss, and the distilled water in each tank was topped up (as appropriate) and recorded.

Tweezers were used to remove leaves, insects, and other foreign matter from the recovered dust tanks. These dust tanks were repeatedly rinsed with distilled water during removal to dislodge any dust on their surfaces. Finally, a broom was used to transfer the dust from the wall of each dust tank to the collection solution. The collection solution of each sample was allowed to evaporate in an evaporating dish, the obtained sample was transferred to a porcelain crucible, and the crucible with the sample was then dried and weighed twice. Prior to use, the porcelain crucibles and empty evaporating dishes were dried in an oven (105 ℃) for 2 h, placed in a dryer to cool, and then weighed twice with a precision electronic balance (Sartorius; precision: 0.01 mg). To correct for the influence of ethylene glycol on the determined results of the dust fall samples, two pure ethylene glycol aqueous solutions were also allowed to evaporate.

During the recovery of each dust tank, the soil under the dust tank, up to a depth of 0–20 cm, was collected. The weight of each soil sample was not less than 300 g. The sampling of the surface soil under each dust tank was performed according to a standard method for the environmental monitoring of soil (HJ/T 166-2004) [26]. After air drying, each collected soil sample was ground in an agate grinding box until it could pass through a nylon sieve (100 mesh). A precision electronic balance was used to weigh 50 g of each ground soil sample, which was then placed in a plastic bag and sealed for storage.

Construction waste samples of concrete, red brick, marble, ceramic tile, glass, wood, plastic, and gypsum from the construction waste dump, were collected simultaneously with the recovery of the dust tanks. The collected construction waste was crushed to a diameter of 2 cm with a hammer and then with a crusher until it could pass through a nylon screen (100 mesh).

#### 2.2.2. Sample Analysis

A precision electronic balance was used to weigh 0.2 g of each dust fall sample (or 0.5 g of each soil or construction waste sample). The sample was then placed in a 50 mL Teflon digestion tank before the successive addition of 6 mL HNO_3_ (Merck, Germany, 65%, Reagent Grade), 2 mL H_2_O_2_ (Merck, Germany, 30%, Reagent Grade), and 0.2 mL HF (Merck, Germany, 40%, Reagent Grade). The prepared samples were subjected to microwave digestion in a digester (MARS 6, CEM Corporation, Matthews, NC, USA). The completely digested dust fall samples were cooled and transferred to a polyester bottle and their volumes were increased to 100 mL with deionised water. The mass fractions of 14 constituent metals, Ca, Fe, Al, Mg, Mn, Zn, Cr, Cu, V, Pb, Ni, As, Co, and Cd, were determined by inductively coupled plasma–mass spectrometry (ICP-MS; Agilent 8800, Tucson, AZ, USA). National marine sediment first-class reference materials GWB07315 and GBW07316, and basalt reference materials BCR-2 and BHVO-2 developed by the U.S. Geological Survey, were used for quality control [27]. Three blank controls were used for each batch of samples. The relative standard deviation and relative error during sample analyses were within ±3%. To correct for any interference from the reagents and ethylene glycol, the average metal contents in the three blanks and two pure ethylene glycol solutions were subtracted from the determined average metal contents in the samples. The stability of the instrument was controlled according to the relative standard deviation (RSD) of the internal standard elements. After each data determination, the relative standard deviation (RSD) of the internal standard elements (45Sc, 73Ge, 115ln, and 209Bi) were checked to ensure an RSD < 3%. The detection limits of 14 metal elements, Ca, Fe, Al, Mg, Mn, Zn, Cr, Cu, V, Pb, Ni, As, Co, and Cd, were 0.0278, 0.0457, 0.0321, 0.0181, 0.0109, 0.0481, 0.0226, 0.0141, 0.049, 0.0120, 0.0128, 0.07, 0.0229, and 0.0031 ppb, respectively. Furthermore, the recoveries of 14 metal elements ranged between 86% and 112%.

### 2.3. Evaluation Method

#### 2.3.1. Enrichment Factor

The enrichment factor (*EF*) is a common metric used to determine the extent to which pollution has increased the presence of an element in the environment [28]. The *EF* is defined as:

(1) $$EF=\frac{{\left(C_{i}/B_{i}\right)}_{\mathrm{sample}}}{{\left(C_{i}/B_{i}\right)}_{\mathrm{background}}}$$

where *C_i_* is the mass fraction of metal *i* (mg/kg), $B_{i}$ is the mass fraction of a reference element (mg/kg), ${\left(C_{i}/B_{i}\right)}_{\mathrm{sample}}$ is the mass fraction ratio of metal *i* to a selected reference element in the dust fall sample, and ${\left(C_{i}/B_{i}\right)}_{\mathrm{background}}$ is the mass fraction ratio of metal *i* to the reference element in the soil (background). In this study, Sc was selected as the reference element, and the arithmetic mean of the mass fraction of each analysed metal in a soil layer of Beijing was selected as the background value of the metal [29]. An *EF* < 10 indicates that the presence of the metal is mainly attributable to natural pollution sources, whereas an *EF* > 10 indicates that it is mainly attributable to man-made pollution sources.

#### 2.3.2. Geo-Accumulation Index

A geo-accumulation index (*I_geo_*) was proposed by German scientist G. Muller in 1969 and is commonly used to describe the degree to which soils and sediments are contaminated with heavy metals. It is also often used to describe the level to which the atmosphere is contaminated with metals [30]. In the present study, the level of metal contamination is classified into seven grades, from no contamination to extremely heavy contamination. The classification standard is shown in Table S1, and *I_geo_* is defined as [31]:

(2) $$I_{geo}=\log_{2}\left[\frac{C_{i}}{k\times B_{i}}\right]$$

where $C_{i}$ is the determined mass fraction of metal *i* in the dust fall sample (mg/kg), $B_{i}$ is the mass fraction of metal *i* in the soil background value; this study adopts the arithmetic mean mass fraction of the metal in a soil layer from Beijing [29], and k is the correction coefficient, which corrects for possible differences in the mass fractions of the metals in the soil (background values) owing to geological differences between regions of 1.5.

#### 2.3.3. Potential Ecological Risk Index

In the ecological risk assessment, the potential ecological risk index considers the category, prevalence, and biological toxicity of pollutants to describe the impact of a single pollutant or the comprehensive impact of multiple pollutants on a specific environment [30]. This index can be used to reflect on not only the level to which heavy metals contaminate the atmosphere, but also the potential ecological risk posed by heavy metals in the dust fall to the environment. It is also currently one of the most common methods used to assess the degree of heavy metal pollution in the environment. In this paper, the potential ecological risk index method proposed in 1980 by Lars Hakanson, a famous Swedish geochemist, was used to evaluate the potential ecological risk of ten toxic heavy metals (Cd, Cu, As, Pb, Ni, Zn, Co, Mn, Cr, and V) in the atmospheric dust around the construction waste dump [32].

(3) $$RI=\sum_{i=1}^{n}E_{i}$$

(4) $$E_{i}=T_{i}\times \left(\frac{C_{i}}{B_{i}}\right)$$

where *RI* is the comprehensive potential ecological risk index, $T_{i}$ is the toxicity coefficient of heavy metal *i* (in this study, the toxicity coefficients of Cd, Cu, As, Pb, Ni, Zn, Co, Mn, Cr, and V are 30, 5, 10, 5, 5, 1, 5, 1, 2, and 2, respectively), $E_{i}$ is the potential ecological risk index of single heavy metal *i*, $C_{i}$ is the determined mass fraction of heavy metal *i* in a dust fall sample (mg/kg), and $B_{i}$ is the mass fraction of heavy metal *i* in the soil (background value; mg/kg).

As the original potential ecological risk classification ignores the situation of no harm, this study reclassifies ‘low risk’ into ‘minor risk’ and ‘low risk’, according to previous studies [33]. The potential ecological risk classification of heavy metals is shown in Table S2.

#### 2.3.4. Grey Correlation Analysis

In the grey correlation analysis, data sequences are translated to geometric shapes that reflect any difference in the data. The closer the geometric shapes of the data sequences, the higher their degree of correlation, and vice versa. This method does not require a high number or wide distribution of samples. Unlike the statistical analysis, grey correlation analysis produces results with directionality, which more accurately reflects the spatial distribution relationship between various factors. Moreover, the results are consistent with those obtained from the quantitative analysis [34,35].

The calculation is shown as follows:

The original sequence is defined as:

(5) $$X_{0}=\left\{x_{0}\left(k\right),k=1,2,\cdots ,n\right\}$$

and we assumed that *m* sequences are to be compared.

(6) $$X_{i}=\left\{x_{i}\left(k\right),k=1,2,\cdots ,n\right\},\;i=1,2,\cdots ,m$$

Then, the grey correlation coefficient ($\xi_{i}\left(k\right)$) is obtained as:

(7) $$\xi_{i}\left(k\right)=\frac{\min\min\left|{\hat{X}}^{\left(0\right)}\left(k\right)-X^{\left(0\right)}\left(k\right)\right|+\rho maxmax\left|{\hat{X}}^{\left(0\right)}\left(k\right)-X^{\left(0\right)}\left(k\right)\right|}{\left|{\hat{X}}^{\left(0\right)}\left(k\right)-X^{\left(0\right)}\left(k\right)\right|+\rho maxmax\left|{\hat{X}}^{\left(0\right)}\left(k\right)-X^{\left(0\right)}\left(k\right)\right|}$$

and the grey correlation degree is evaluated as

(8) $$r_{i}=\frac{1}{n}\sum_{k=1}^{n}\xi_{i}\left(k\right)$$

where *k* = 0, 1, …, *n*, ${\hat{X}}^{\left(0\right)}\left(k\right)$ is the reference data sequence, which is the heavy metal content in the atmospheric dust, $x_{0}\left(k\right)$ is the data sequence used for comparison, and in this study, it is the metal content in the surface soil, and *ρ* is the resolution coefficient, often considered to be 0.5.

## 3. Results

### 3.1. Metal Contents in the Dust Fall in the Vicinity of a Construction Waste Dump in Beijing

The metal (Ca, Al, Fe, Mg, Mn, Zn, Cr, Cu, V, Pb, Ni, As, Co, and Cd) contents (mean, coefficient of variation, sample-to-background ratio, and sample-to-control point ratio) in the dust fall samples obtained at selected points in the vicinity of a construction waste dump in Beijing are shown in Table 1. The sample-to-background ratio is the ratio of the determined metal content in the dust fall sample to that in the soil (background value) in Beijing, whereas the sample-to-control point ratio is the ratio of the determined metal content in the dust fall sample to that in the control sample.

Table 1 shows that the average metal contents in the dust fall decrease in the order Ca > Fe > Al > Mg > Mn > Zn > Cr > Cu > V > Pb > Ni > As > Co > Cd. In this study, the sample-to-background ratios of Ca, Fe, Mg, Mn, Zn, Cd, and Cu exceeded one, indicating that the dust fall contains higher amounts of these seven metals than the soil in Beijing. The sample-to-background ratio of Cd (4.41) is the highest, followed by those of Ca and Zn. The sample-to-control point ratios of the 14 metals exceeded one. These results suggest that the construction waste dump contributes to air pollution in the area. Moreover, the sample-to-control point ratio of Cd was the highest among the metals, indicating that Cd is a significant atmospheric pollutant. The coefficients of variations of Ca, Mn, Zn, Cr, Cu, V, Pb, Ni, As, and Co were <0.5, indicating that the spatial distribution of these ten metals did not vary significantly. In contrast, the coefficients of variations of Fe, Al, Mg, and Cd were 0.70, 0.77, 0.56, and 0.65, respectively. This indicates that there is local pollution of Fe, Al, Mg, and Cd in the study area.

The Ca, Fe, Al, and Mg contents in the dust fall were high, accounting for 98.81% of the total mass of the analysed metals, wherein Ca accounted for 45.66% of the total mass of the analysed metals. The remaining ten toxic heavy metals accounted for less than 2% of the total mas of the analysed metals, of which As, Co, and Cd accounted for only 0.005%, 0.004%, and 0.001%, respectively, of the total mass of the analysed metals.

### 3.2. Enrichment Factors of Metals in the Dust Fall in the Vicinity of the Construction Waste Dump

Ren et al. [10] discussed the relationship between the dust fall amount and the distance of the construction waste dump. The results showed that the dust fall amount was negatively correlated with the distance, and the correlation was significant when the distance was less than 500 m. Therefore, when the characteristics of heavy metal pollution in the dust fall of construction waste are analysed, the sampling points are divided into three categories (<250 m, 250–500 m, 500–1000 m) according to the distance.

The *EFs* of 14 metals present in the dust fall in the vicinity of the construction waste dump are shown in Table S3. The *EFs* of Mn, Fe, Pb, Cr, As, Ni, V, Co, Mg, and Al at all sampling points were <10, indicating that the presence of these ten metals in the dust fall was mainly attributable to natural pollution sources within a radius of 1 km of the construction waste dump. The *EFs* of Cu at sampling points 7 and 16 were >10, indicating that the presence in the dust fall at these locations was mainly attributable to man-made pollution sources, whereas its presence at the other 16 sampling points was mainly attributable to natural pollution sources. The presence of Cd and Ca in the dust fall was mainly attributable to man-made pollution sources within 250 m of the dump, natural pollution sources between 250 and 500 m from the dump, and man-made pollution sources between 500 and 1000 m from the dump. The *EFs* of Cd at sampling points 14, 15, and 18 (which were farther than 500 m from the dump) were high, indicating that the significant presence of Cd at these three sampling points was attributable to man-made pollution sources. Figure S1 shows that these sampling points are mainly located near roads. According to the field survey, there are no other artificial pollution sources within 500 m of the study area except construction waste, and no industrial pollution sources within 5 km. Therefore, Cd within 250 m of the construction waste dump is mainly affected by construction waste. Cd within 500–1000 m of construction waste dump is mainly from road pollution sources. The presence of Zn and Cu in the dust fall within 1 km of the dump was mainly attributable to natural pollution sources. The *EFs* of the analysed metals in the dust fall at the control point were <10, indicating that their presence was mainly attributable to natural pollution sources.

### 3.3. Pollution of Heavy Metals in Dust Fall in the Vicinity of a Construction Waste Dump

The *I_geo_* values determined for ten heavy metals in the dust fall in the vicinity of the construction waste dump are shown in Table 2. The *I_geo_* values of the ten heavy metals in the dust fall at the control point were <0, corresponding to a grade of contamination of 0, thus implying no contamination. The *I_geo_* values of the heavy metals in the dust fall varied with the distance (0–1 km) from the dump. However, regardless of the distance, the *I_geo_* values of Cd, Zn, and Cu were >0, with contamination grades of 1–3, indicating a certain level of contamination. Within 500 m of the construction waste dump, there is moderate contamination of Zn, light to moderate contamination of Cd, and light contamination of both Mn and Cu. In addition, certain levels of Mn contamination were detected within 500 m of the dump. The *I_geo_* values of Pb, Cr, As, Ni, V, and Co in the dust fall, regardless of distance from the dump, were less than 0, corresponding to a contamination grade of 0, thus implying no contamination. In general, the prevention and control of heavy metal pollution caused by atmospheric dust in the construction waste dump should focus on the monitoring of Cd, Zn, and Cu.

### 3.4. Potential Ecological RISK Assessment of Heavy Metals in the Dust Fall in the Vicinity of the Construction Waste Dump

#### 3.4.1. Potential Ecological Risk Level of Heavy Metals

The $E_{i}$ values of ten toxic heavy metals, Cd, Cu, As, Pb, Ni, Zn, Co, Mn, Cr, and V, in the dust fall in the vicinity of the construction waste dump are shown in Table 3. The $E_{i}$ values of the ten heavy metals in dust fall within 500 m of the dump decreased in the order Cd > Cu > As > Pb > Ni > Zn > Co > Mn > Cr > V. Moreover, the $E_{i}$ values of the ten heavy metals in dust fall between 500 and 1000 m from the dump decreased in a similar order, except for the $E_{i}$ of Mn, which exceeded that of Co. The $E_{i}$ values of Cd in the dust fall within 250 m of and between 250 and 500 m from the storage dump were 127.13 and 89.52, respectively, indicating considerable ecological risk. In contrast, the $E_{i}$ value of Cd in the dust fall between 500 and 1000 m from the dump was 191.28, indicating high ecological risk. The $E_{i}$ values of As, Pb, Ni, Zn, Co, Mn, Cr, and V in the dust fall decreased (in terms of distance from the dump) in the order (<250 m) > (250–500 m) > (500–1000 m). However, all the $E_{i}$ values of these heavy metals were <10, indicating that they only posed minor ecological risks. The comprehensive potential ecological risk (*RI*) of the ten heavy metals in the dust fall decreased (in terms of distance from the dump) in the order (500–1000 m) > (<250 m) > (250–500 m). The comprehensive potential ecological risks posed by these metals in the dust fall within 250 m of and between 500 and 1000 m from the dump were moderate, whereas the corresponding comprehensive potential ecological risk between 250 and 500 m from the dump was low.

#### 3.4.2. Distribution of the Comprehensive Potential Ecological Risk Posed by Heavy Metals in the Dust Fall in the Vicinity of the Construction Waste Dump

**Figure 1:**
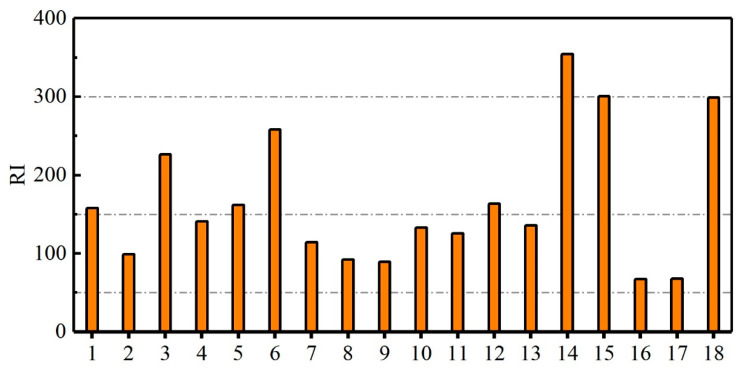
*RI* values of heavy metals in dust fall at various sampling points in the vicinity of the construction waste dump.

The *RI* values of the heavy metals in the dust fall at each sampling point in the vicinity of the construction waste dump are shown in Figure 1. The *RI* values of the heavy metals in the dust fall at the 18 sampling points (excluding the control point) were in the range of 67.46–354.49. Except for the *RI* value at sampling point 14, the *RI* values of the heavy metals in the dust fall did not exceed 300, indicating that the comprehensive ecological risk posed by the heavy metals was low to moderate. The *RI* values of the heavy metals in the dust fall at seven sampling points were between 150 and 300, indicating moderate ecological risk, whereas the *RI* values of the heavy metals in the dust fall at the other ten sampling points were between 50 and 150, indicating low ecological risk. The highest *RI* value (>300) of the heavy metals in the dust fall was obtained at sampling point 14, indicating high comprehensive ecological risk, whereas the lowest *RI* value was obtained at sampling points 16 and 17, indicating low comprehensive ecological risk. Figure S1 shows that sampling point 14 is near a main road with high traffic flow, suggesting that the heavy metals in the dust fall mainly originated from road pollution sources, thus explaining the high comprehensive ecological risk that was determined. Sampling points 16 and 17 are located in a park to west of the construction waste dump, and the park is a significant distance away from the construction waste dump and road pollution sources; therefore, the heavy metals in the dust fall at these two sampling points pose low ecological risks.

**Figure 2:**
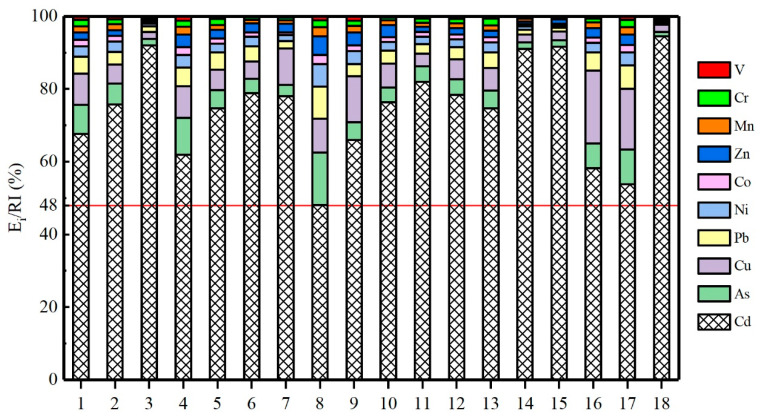
Contributions of respective heavy metals to the comprehensive ecological risk posed by these metals at various sampling points in the vicinity of the construction waste dump.

Figure 2 shows that Cd has the highest contribution (>48%) to the *RI* value of the heavy metals, and Cu, As, and Pb also contribute significantly. In contrast, V, Mn, and Cr have the lowest contribution to the *RI* value of the heavy metals. In conjunction with the results of the ecological risk assessment (Section 3.4.1), these results indicate that Cd is the primary ecological risk factor in the dust fall in the vicinity of the construction waste dump.

### 3.5. Correlation between the Metal Contents in the Topsoil and Dust Fall in the Vicinity of the Construction Waste Dump

The correlations between the metal contents in the surface soil and dust fall in the vicinity of the construction waste dump are shown in Table 4. The degree of correlation between the metal contents in the surface soil and dust fall is >0.6, indicating a significant correlation. The degree of correlation between the metal contents in the topsoil and dust fall decreases in the order Ca > Al > V > Co > Cd > Mn > Ni > Fe > Mg > Cu > As > Cr > Zn > Pb. The degree of correlation of Ca of 0.78 is only slightly higher than that of Al of 0.77, and the degrees of correlation of the other metals in the sequence are similarly high. The degree of correlation of Pb is the lowest, indicating that the Pb contents in the topsoil and dust fall had the highest difference.

## 4. Discussion

The results of this study show that the average metal contents in the dust fall in the vicinity of the construction waste dump decrease in the order Ca > Fe > Al > Mg > Mn > Zn > Cr > Cu > V > Pb > Ni > As > Co > Cd. The content sequence of Pb, Ni, As, and Cd is consistent with the research results obtained by Wang et al. [36] regarding the metal elements in dust fall during building demolition (Pb > Ni > As > Cd in dust fall). The research results obtained by Nhien and Giao [37] regarding the metal elements pollution of Cai Dau landfill in Vietnam show that the concentrations of Fe and Mn are the highest, followed by Zn and Cr, and finally Cu, Ni, Pb, and As. In addition, similar to the results obtained by Lei et al. [38], the contribution rate of Ca, Fe, Al, and Mg to the building dust is very high. The research results obtained by Vega et al. [39] show that Ca and Fe are typical characteristics of most geological materials, while cement and crushed gravel also contains an abundance of Ca. There were many cement fragments, gypsum boards, and crushed gravels in the construction waste dump selected in this paper; therefore, the average content of Ca is higher than other elements.

Cd, Zn, Cu, and Mn in the dust fall ranged from slight to severe. Pb, Cr, As, Ni, V, and Co in the dust fall implies no contamination. The analysis results regarding the heavy metals in the construction waste soil obtained by Wu et al. [40] showed that Cd, Zn, Pb, and Cu are characteristic pollutants of construction waste in landfills. Other studies show that in other countries in the world (such as Malaysia, Philippines, Laos, and Iran), Zn and Cr are the main heavy metal pollutants in the construction waste landfill soil [41,42]. The evaluation results regarding the *I_geo_* values of heavy metals in the soil of a Tibetan landfill site show that Ni, Cr, and As have zero to moderate contamination [43]. In addition, the results regarding the *I_geo_* values of heavy metals in the soil of mixed landfill (construction waste and domestic waste) obtained by Nhien and Giao [37] also show that Ni, Cr, and As have high, moderate to high, and zero to moderate contamination, respectively. However, in this study, Cr in the dust from the construction waste dump poses almost no harm to the atmospheric environment. The reason for this difference may be that the garbage dump in this study only consists of construction garbage, and there is no pollution from domestic garbage. The $E_{i}$ values of Cd in the dust fall within 500 m of the storage dump indicated considerable ecological risk. The $E_{i}$ values of As, Pb, Ni, Zn, Co, Mn, Cr, and V in the dust fall were <10, indicating that they posed minor ecological risks. However, the results obtained by Wang et al. [41] show that the ecological risk of soil Cr in different types of landfill sites is high. It can be seen that, in the prevention and control of construction waste pollution in Beijing, the heavy metal elements, such as Cd, Zn, and Cu, in the site should be monitored.

During the long-term storage and associated weathering of stacked construction waste, toxic and harmful heavy metals contained in the construction waste are dispersed in the air with other dust particles, thus polluting the soil in the vicinity of the construction waste dump. This phenomenon is supported by the correlation determined between the metal contents in the topsoil and dust fall in the vicinity of the construction waste dump. The differences observed in the degrees of correlation of the various metals indicate that their accumulation in the topsoil varies, which is attributable to differences in their sedi-mentation behaviour [44]. In addition, Castillo et al. [45] studied the atmospheric deposition of wastes in the Rio Tinto mining area in Spain. The results showed that the mining wastes have potential adverse effects on the surrounding soil, plants, and human beings. The different types of construction waste found in the construction waste dump contained the following metals: Ca, Fe, Al, Mg, Mn, Cr, Cu, V, Pb, Ni, As, and Co (Table S4). Ca is a characteristic element of construction waste and is present in the concrete, marble, glass, wood, plastic, and gypsum found in the construction waste dump. The Ca contents in the marble, gypsum, and concrete exceeded that in the dust fall in the vicinity of the dump by factors of 6, 3, and 2, respectively. Zn was detected in all the analysed types of construction waste, except marble. Moreover, the Zn content in the glass was similar to that in dust fall in the vicinity of the construction waste dump, but exceeded that of soil (background value) in the vicinity of the dump by a factor of 3. Cd was detected in all the analysed types of construction waste, except concrete. Furthermore, the Cd content in the gypsum board was as high as 0.32 mg/kg, which exceeded that in the soil (background value) and dust fall in the vicinity of the construction waste dump by a factor of four. The Zn and Cu contents in the glass were 356.14 and 96.53 mg/kg, respectively. Yu et al. [46] also showed that the content of Cd in gypsum board was higher than that of other types of construction waste; however, the research also shows that the content of Zn in ceramic tiles is 596.72 mg/kg, which is much higher than that in this study. The content of Cu in eight kinds of construction waste is lower than the background value of soil and atmospheric dust in Beijing. Gao et al. [47] measured and analysed heavy metals in different building demolition waste samples and the results showed that Zn pollution in residential waste samples was serious. Generally, the total experimental results from different construction waste types and previous study results show that there are heavy metals in construction waste.

While the results are helpful to understand pollution dynamics of atmospheric dust around a construction waste site, they could also provide a scientific basis for the management and control of construction waste. Due to study limitations, the heavy metal Hg was not determined in this paper. Hg will need to be included in a follow-up study. In addition, the source of each metal element in the atmospheric dust fall should be quantitatively analysed. Future studies should quantitively investigate the sources of various metals in the atmospheric dust to inform the sustainable management and storage of construction waste.

## 5. Conclusions

We investigated the composition of 14 metal elements in the atmospheric dust fall of a large construction waste dump in Beijing, and further studied the pollution dynamics of 10 heavy metals and their potential ecological risks to the surrounding environment. The results show that Ca, Fe, Al, and Mg contents were found to be considerably high in the dust fall in the vicinity of the construction waste dump selected in this study. These four metals accounted for 98.81% of the total mass of the analysed metals in the dust fall. Cd and Zn were determined to be the primary metal contaminants in the dust fall in the vicinity of the construction waste dump, followed by Cu and Mn. The potential ecological risk assessment shows that the individual ecological risks posed by the Cu, As, Pb, Ni, Zn, Co, Mn, Cr, and V in the dust fall were minor; however, Cd had the highest contribution to the comprehensive ecological risk posed by heavy metals and was determined to be the primary ecological risk factor in the atmospheric environment in the vicinity of the construction waste dump. A strong correlation was obtained between the metal contents in the topsoil and dust fall in the vicinity of the construction waste dump, indicating that the metal contents in the dust fall had a significant impact on the metal contents in the topsoil. Our results are significant within the context of constructing “waste free cities” in China and provide a robust reference for future research on such construction sites in other cities.

## Figures and Tables

**Table 1:** Metal contents in dust fall in the vicinity of a construction waste dump in Beijing.

Element	Average Content (Unit: mg/kg)	Sample-To-Background Ratio	Sample-To-Control Point Ratio	Coefficient of Variation
Ca	67,123.5	4.42	1.74	0.20
Fe	36,411.3	1.23	1.63	0.70
Al	22,522.4	0.32	2.83	0.77
Mg	19,223.7	1.51	1.42	0.56
Mn	1250.14	1.77	1.84	0.36
Zn	308.97	3.01	2.15	0.47
Cr	53.06	0.78	1.55	0.46
Cu	43.15	1.83	1.23	0.32
V	33.56	0.42	1.47	0.43
Pb	25.25	0.99	1.35	0.47
Ni	19.43	0.67	1.67	0.42
As	6.82	0.7	1.41	0.47
Co	6.04	0.36	1.53	0.37
Cd	0.33	4.41	3.5	0.65

**Table 2:** Geo-accumulation index (*I_geo_*) values of heavy metals in the dust fall in the vicinity of the construction waste dump.

Distance	Element	Cd	Zn	Mn	Cu	Pb	Cr	As	Ni	V	Co
<250 m	*I_geo_* value	1.5	1.19	0.4	0.38	−0.32	−0.68	−0.84	−0.98	−1.63	−1.86
	Grade of contamination	2	2	1	1	0	0	0	0	0	0
	Contamination characterisation	Moderate	Moderate	Light	Light	*	*	*	*	*	*
250–500 m	*I_geo_* value	0.99	1.11	0.3	0.16	−0.56	−0.83	−1.09	−1.04	−1.7	−2.03
	Grade of contamination	1	2	1	1	0	0	0	0	0	0
	Contamination characterisation	Light	Moderate	Light	Light	*	*	*	*	*	*
500–1000 m	*I_geo_* value	2.09	0.53	−0.09	0.29	−1.14	−1.64	−1.54	−1.67	−2.35	−2.52
	Grade of contamination	3	1	0	1	0	0	0	0	0	0
	Contamination characterisation	Moderate to severe	Light	*	Light	*	*	*	*	*	*
Control point	*I_geo_* value	−0.25	−0.1	−0.64	−0.02	−1.02	−1.58	−1.59	−1.9	−2.38	−2.69
	Grade of contamination	0	0	0	0	0	0	0	0	0	0
	Contamination characterisation	*	*	*	*	*	*	*	*	*	*

Note: * indicates no contamination.

**Table 3:** Potential ecological risk of heavy metals in the dust fall in the vicinity of the construction waste dump.

Distance	<250 m		250–500 m		500–1000 m	
Element	$E_{i}$ Value	Potential Ecological Risk Level	$E_{i}$ Value	Potential Ecological Risk Level	$E_{i}$ Value	Potential Ecological Risk Level
Cd	127.13	Considerable	89.52	Considerable	191.28	High
Cu	9.78	Minor	8.40	Minor	9.15	Minor
As	8.37	Minor	7.05	Minor	5.16	Minor
Pb	6.00	Minor	5.07	Minor	3.41	Minor
Ni	3.80	Minor	3.66	Minor	2.35	Minor
Zn	3.41	Minor	3.25	Minor	2.17	Minor
Co	2.06	Minor	1.85	Minor	1.30	Minor
Mn	1.97	Minor	1.84	Minor	1.41	Minor
Cr	1.87	Minor	1.69	Minor	0.96	Minor
V	0.97	Minor	0.92	Minor	0.59	Minor
*RI*	165.36	Moderate	123.25	Low	217.78	Moderate

**Table 4:** Correlation between the metal contents in the topsoil and dust fall in the vicinity of the construction waste dump.

Element	Ca	Al	V	Co	Cd	Mn	Ni	Fe	Mg	Cu	As	Cr	Zn	Pb
*r*	0.78	0.77	0.75	0.73	0.73	0.71	0.70	0.69	0.68	0.68	0.65	0.64	0.63	0.61

## Data Availability

The data supporting this study’s findings are available from the first author (L.W.) upon reasonable request.

## Supplementary Materials

The following supporting information can be downloaded at: https://www.mdpi.com/article/10.3390/ijerph192013019/s1, Figure S1: Study area and distribution of dust fall sampling points; Table S1: Correspondence between the level of contamination and geo-accumulation index; Table S2: Potential ecological risk classification; Table S3: Enrichment factors of various metals found in dust fall in the vicinity of the construction waste yard; Table S4: Metal contents of different types of construction waste.
