# Supplementary material for: Characterisation and Risk Assessment of Metal Contaminants in the Dust Fall in the Vicinity of a Construction Waste Dump in Beijing
Source: Int J Environ Res Public Health. 2022 Oct 11;19(20):13019. doi: 10.3390/ijerph192013019 (PMC9602486; doi:10.3390/ijerph192013019)
Supplement: Supplementary file 1 [file ijerph-19-13019-s001.zip › ijerph-1933327-supplementary.pdf]

Supplementary Material

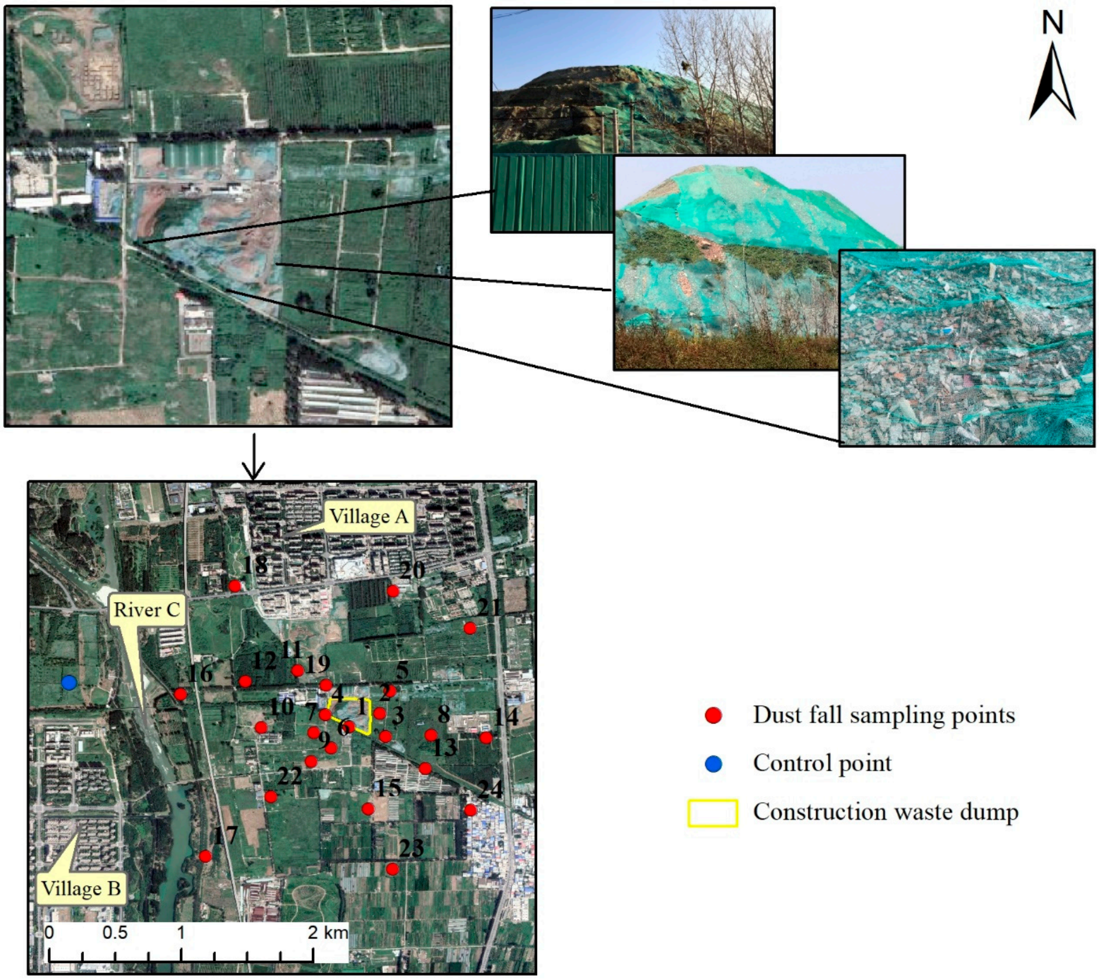

Figure S1. Study area and distribution of dust fall sampling points

Table S1. Correspondence between the level of contamination and geo-accumulation index

Grade	I_{geo}	Level of contamination
0	≤0	No contamination
1	0–1	Light contamination
2	1–2	Moderate contamination
3	2–3	Moderate to severe contamination
4	3–4	Severe contamination
5	4–5	Severe to extremely heavy contamination
6	>5	Extremely heavy contamination

Table S2. Potential ecological risk classification

E_i	Individual potential ecological risk level	RI	Comprehensive potential ecological risk level
E_i<10	Minor risk	RI<50	Minor risk
10≤E_i<40	Low risk	50≤RI<150	Low risk
40≤E_i<80	Moderate risk	150≤RI<300	Moderate risk
80≤E_i<160	Considerable risk	300≤RI<600	High risk
160≤E_i<320	High risk	RI≥600	Extremely high risk
E_i≥320	Extremely high risk	/	/

Note: / indicates no data

Table S3. Enrichment factors of various metals found in dust fall in the vicinity of the construction waste yard

Distance	Number	Cd	Ca	Zn	Cu	Mn	Fe	Pb	Cr	As	Ni	V	Co	Mg	Al
	1	10.56	14.52	9.77	8.06	7.87	7.74	4.30	4.13	3.74	2.72	2.24	1.68	1.50	0.34
	2	5.14	8.41	3.19	2.10	3.37	2.79	1.44	1.42	1.19	1.13	0.84	0.64	6.00	1.36
	3	19.64	10.21	3.04	2.47	2.52	0.93	1.89	1.31	1.12	1.01	0.86	0.63	9.64	0.56
＜250 m	4	5.25	11.18	8.92	4.47	5.36	5.12	2.63	2.23	2.58	1.74	1.49	1.07	1.63	1.14
	5	7.13	6.23	6.89	3.26	3.66	3.72	2.71	2.44	1.45	1.41	1.04	0.79	3.33	1.41
	6	17.46	13.58	16.56	6.38	6.55	0.81	5.56	2.60	2.64	3.54	0.73	1.46	3.06	0.53
	7	13.22	20.47	12.12	10.25	4.77	1.21	2.03	1.73	1.60	1.64	1.05	0.80	2.66	0.51
	8	4.51	8.07	14.24	5.24	7.06	5.90	4.97	2.75	4.09	3.48	1.55	1.43	2.53	0.47
	9	4.50	10.39	7.41	5.14	3.70	3.24	1.40	1.50	1.01	1.45	1.20	0.61	2.32	0.40
250-500 m	10	12.94	21.35	16.98	6.73	6.79	1.18	3.67	1.76	2.08	2.41	1.10	1.26	5.07	0.73
	11	5.73	4.93	2.91	1.48	2.45	2.37	1.11	1.21	0.89	0.84	0.65	0.53	1.16	1.03
	12	9.01	8.96	6.17	3.77	4.16	4.55	2.27	2.18	1.47	1.55	1.26	0.90	1.41	1.15
	13	6.42	10.60	4.69	3.20	3.65	2.41	2.18	2.45	1.27	1.42	0.86	0.75	4.90	1.40
	14	40.70	16.49	10.50	5.65	9.88	6.68	3.42	2.63	2.39	2.71	1.79	1.51	7.10	0.51
500-1000 m	15	24.83	11.83	9.44	3.95	3.06	0.95	1.62	1.27	1.40	1.14	0.85	0.70	4.85	0.53
	16	3.84	17.19	6.81	10.64	4.09	0.99	2.70	1.44	1.80	1.38	0.87	0.79	5.51	0.45
	17	3.49	16.32	7.31	8.68	5.40	4.80	3.30	2.59	2.48	1.91	1.30	1.04	4.09	0.44

18	47.49	22.47	4.81	6.20	4.00	0.71	1.81	1.05	1.66	1.83	0.65	0.88	13.16	0.47
Control point	4.90	9.86	5.45	6.10	3.75	2.92	2.87	1.95	1.94	1.56	1.12	0.90	4.15	0.44

Table S4. Metal contents of different types of construction waste

Element	Concrete	Red brick	Marble	Ceramic tile	Glass	Wood	Plastic pipe	Plaster
Ca	131935.42	10863.19	372751.25	13751.89	58328.97	19412.62	169600.14	219827.75
Fe	24432.84	5917.31	2475.31	7039.50	7247.48	1544.86	2482.61	1608.76
Mg	16112.81	2042.28	2571.64	4807.30	20954.18	2756.73	8788.69	92600.57
Al	11106.40	6026.37	653.29	6615.84	4596.24	1298.51	1983.13	1753.62
Cr	842.81	11.03	30.42	901.55	1669.19	16.61	70.30	4.72
Mn	532.72	141.39	330.76	127.01	114.75	56.23	105.63	97.22
V	54.36	11.70	1.24	12.75	12.36	4.70	8.63	4.34
Ni	23.90	1.79	2.53	22.24	167.24	2.43	12.08	0.57
Zn	17.91	1.49	0.00	31.73	356.14	37.09	26.86	3.79
Pb	8.48	5.04	2.29	4.54	12.64	2.68	3196.61	2.02
Cu	6.48	2.46	0.04	2.20	3.53	6.11	2.16	1.44
Co	5.61	1.84	0.73	2.36	6.44	0.80	1.02	0.69
As	4.49	4.60	1.90	3.65	2.87	2.04	2.54	2.38
Cd	0.00	0.01	0.03	0.02	0.03	0.28	0.19	0.32
